# The influence of sex on sleep characteristics in the adult and older adult population: findings from the EPISONO sleep study

**DOI:** 10.3389/frsle.2025.1422169

**Published:** 2025-02-10

**Authors:** Mayra dos Santos Silva, Priscila Farias Tempaku, Monica Levy Andersen, Sergio Tufik, Dalva Poyares

**Affiliations:** ^1^Departamento de Psicobiologia, Universidade Federal de São Paulo, São Paulo, Brazil; ^2^Sleep Institute, São Paulo, SP, Brazil

**Keywords:** sex, sleep, polysomnography, adult, older adults

## Abstract

**Introduction:**

Sleep is a restorative behavior and is critical for overall health, but differences in sleep patterns between men and women can be seen over the years.

**Aim:**

To prospectively analyze the polysomnographic findings of adults and older adults who participated in two editions of EPISONO, according to sex.

**Methods:**

The population-based prospective longitudinal study included 688 individuals. Of these, there were 389 women and 299 men. All examinations and tests were undertaken using the same protocols at both times, at baseline and 8 years later. All participants completed an institutional questionnaire and a range of other questionnaires on health and sleep parameters. They also underwent full-night polysomnography (PSG) and peripheral blood collection for biochemical and hematological measurements. In both editions (2007 and 2015), physical and anthropometric assessments were also assessed. Exclusion criteria: pregnant or lactating women, individuals with self-care limitations (physical or mental), and night-shift workers.

**Results:**

We observed that adult women showed a greater REM latency, adult and older adult women spent more time in N3 than men, and an increased periodic leg movement index in older adult women. Men (adults and older adults) remained longer in N1, had a higher number of awakenings, and of apnea-hypopnea index. Age was shown to be an influencing factor for changes in arousal index and apnea-hypopnea index. This study prospectively evaluated, with full-night PSG, the sleep of the general population and reported significant findings according to sex and age, suggesting that some sleep parameters change differently in men and women, as we age.

## 1 Introduction

Men and women have different sleep characteristics and behaviors. Studies have shown that women experience longer total sleep time (TST), more slow wave sleep (SWS), and shorter rapid eye movement (REM) sleep duration than men (Morssinkhof et al., 2023). A systematic review with 3,712 articles showed that male participants had reduced REM latency, a higher arousal index, and apnea-hypopnea index (AHI; Boulos et al., 2019). A study that recorded the polysomnography (PSG) of a group of 31 healthy volunteers, with an average age of 20 years old, reported that women had significantly longer TST, shorter sleep onset latency, and better sleep efficiency than men (Goel et al., 2005).

Similarly, a cross-sectional analysis of portable PSG measurements of 2,685 middle-aged participants found that men had lighter sleep compared to age-matched women (Redline et al., 2004). Specifically, men had a higher percentage of N1 and N2 stage sleep and a lower percentage of SWS and REM sleep. Despite these findings pointing out that healthy women objectively have better sleep quality than men, there is a paradox, as women across a wide age range tend to mention more sleep problems than men in subjective studies that use self-assessment (Reyner et al., 1995; Zhang and Wing, 2006). They complained of worse sleep quality, difficulties falling asleep, frequent nocturnal awakenings, and longer periods awake throughout the night (Lindberg et al., 1997). However, the reasons for this discrepancy between subjective and objective sleep findings remain unclear.

Regarding sleep disorders, women are twice as likely to experience sleep disruption and insomnia throughout their lives as men (Smith and Mong, 2019). The most likely explanations for this are the hormonal fluctuations that occur across a woman's life and the fact that women seek help for sleep problems more often than men (Bailey and Silver, 2014). Recent clinical studies have shown that women who suffer from sleep disorders and insufficient sleep are at a higher risk than men developing mood disorders, such as depression (Krystal, 2004), metabolic (Lyytikäinen et al., 2011), and cardiovascular dysfunctions (Ferrie et al., 2007). In addition to sleep interruptions and insomnia, a study of restless legs syndrome suggested that there was a higher prevalence of the condition in women (Unruh, 1996; Vallerand and Polomano, 2000).

In contrast, men tend to have more obstructive sleep apnea (OSA). A German study with a population aged between 20 and 80 years old showed that the prevalence of OSA increased continuously with age for men and women, but started later in life for women. The same study found that there was a significant, positive association between OSA and male sex, age, body mass index (BMI), waist-to-hip ratio, snoring, alcohol consumption, and self-reported cardiovascular disease (Fietze et al., 2019).

Study with 470 subjects enrolled in the Sleep Heart Health Study revealed clear lessening in the quantity and quality of sleep with age that appears to be more rapid in males compared to females (Walsleben et al., 2004). Alterations in sleep architecture, characterized by changes in the quantity and quality of sleep as well as in sleep stages and duration, generally becomes present as we advance into the 5^th^ decade of life (Mander et al., 2017). A systematic review showed that with each decade of age, total sleep time and sleep efficiency decreased, wake-after sleep onset and arousal index increased (Boulos et al., 2019). Although REM sleep appears to be preserved with age, the amount of SWS may decrease (Ehlers and Kupfer, 1997; Hume et al., 1998; Reynolds et al., 1991). Older women tend to wake up earlier than desired and have a higher rate of sleep complaints compared to men (Doghramji, 2006). but the reasons for these observed differences are not well-elucidated.

The literature presents conflicting findings regarding sleep changes with aging, across sexes, likely influenced by the use of non-standardized or different methodologies. This study specifically highlights differences in sleep patterns between males and females based on polysomnographic findings from adults and older adults who participated in the Episono study. Notably, this is one of the few studies to prospectively evaluate sleep in the general population using comprehensive in-laboratory polysomnography while reporting sex-specific differences.

## 2 Materials and methods

### 2.1 Studied sample

This study is part of a population-based survey with a longitudinal design of the individuals from the São Paulo Epidemiologic Sleep Study (EPISONO) cohort (baseline: 2007), which was followed over 8 years (follow-up: 2015). The sample used in the baseline study was representative of both sexes (women and men), age groups (20 to 80 years old), and social classes of the city. The exclusion criteria were pregnant and nursing women, shift workers and people with self-care limitations (physical or mental). All volunteers originally included in the baseline study were invited by telephone to revisit the sleep laboratory to undergo the same analyzes performed in the baseline study. The project was approved by the Research Ethics Committee of the Universidade Federal de São Paulo (CEP 0593/06); (2014/610.514) and registered at ClinicalTrials.gov (Identifier NCT00596713).

The cluster randomized sample at baseline (2007) included 1,042 volunteers, but in 2015, of these individuals in the EPISONO cohort, 327 were excluded (refused to participate, they were not located, or they died), ending with 715 individuals (Piovezan et al., 2019).

Our particular study analysis was conducted as a subset of the EPISONO cohort, considering the individuals who participated in both baseline and follow-up data—individuals who answered all the questionnaires of interest, completed all physical assessments, and underwent in-lab full polysomnography. Finally, we used a sample composed of 688 participants, 389 women and 299 men.

### 2.2 Questionnaires

The following questionnaires were used:

- Pittsburgh Sleep Quality Index (PSQI) is a self-rated questionnaire comprising 19 questions that assesses sleep quality and disturbances over 1 month and provides a sleep quality score (Buysse et al., 1989);
- Berlin Questionnaire is a brief questionnaire with ninie questions to assess the risk for OSA (Netzer et al., 1999);
- Epworth Sleepiness Scale (ESS) comprises eight questions that measure a person's likelihood of falling asleep or dozing off in different situations commonly encountered in daily life, thus assessing daytime sleepiness (Lapin et al., 2018).

### 2.3 Polysomnography

All individuals were subjected to a full-night PSG at baseline and in the follow-up studies at the sleep laboratory using the EMBLA^®^ N7000 system (Embla Systems Inc., USA). Physiological variables evaluated during PSG included: electroencephalogram (6 channels at follow-up vs. 4 channels at baseline); electrooculogram (2 channels); surface electromyogram (4 channels); electrocardiogram (1 channel: derivation D2 modified at follow-up vs. derivation V1 modified at baseline); air flow (2 channels); respiratory effort (2 channels) by inductance plethysmography belts; snoring and body position (1 channel each) by EMBLA^®^ sensors; and oxygen saturation (SpO2) by EMBLA^®^ oximeter. Both baseline and follow-up studies were performed according to the standardized criteria of the American Academy of Sleep Medicine (AASM) recommended setup specification and all 2007 recordings were revised according to current scoring manual used in 2015 (Iber et al., 2007). Hypopneas were scored according to the recommended rule of the 2012 AASM manual, i.e., a decrease of 30% in the respiratory flow associated with an arousal or 3% oxygen desaturation (Berry et al., 2013). Sleep efficiency was considered decreased when it fell below 85%, and the arousal index was considered normal when there were fewer than 10 events per hour. An objective polysomnographic diagnosis of OSA was defined when the AHI was greater or equal to 5 events per hour. Also, OSA was evaluated considering its severity by AHI values: non-AHI to mild AHI (5–14.9 events/hour); moderate AHI (15–29.9 events/hour); and severe AHI (AHI > = 30).

The occurrence of periodic leg movements (PLM) when there were 15 or more events per hour. As the sample covered a wide age range, we allocated the volunteers to adults (aged up to 59 years old) and older adults (over 60 years old) and named those participants who were in the range between 52 and 59 years old at baseline but who turned over 60 years old in the reassessment in 2015/2016 as new older adults.

### 2.4 Statistical analysis

The descriptive analyses were performed to characterize the profile of the studied sample in relation to age, sex, BMI, objective PSG parameters and subjective sleep parameters. These analyses were described using means and standard deviations, or counts and percentages. One-way ANOVA or Kruskal–Wallis and χ2 tests of homogeneity, or Fisher's exact test were used when appropriate to compare participants' characteristics. The Generalized Estimating Equations (GEE) test was applied to compare Baseline (2007) and follow-up (2015) moments. For the analysis of parameters measured at a single point of time, the Generalized Linear Models (GLzM) test was used. The analysis of the association of categorical variables for each period was tested with the Chi-square test. For significant effects of variables with more than two categories, a Bonferroni *post hoc* test was performed. Analyzes were performed using the PASW Statistics 20 (SPSS Inc., USA) statistical package. A significance level of 0.05 was considered, with 95% statistical confidence.

## 3 Results

We performed descriptive analysis only for individuals who were followed in 2015 and fulfilled the inclusion criteria (*n* = 688). We noticed a small increase in BMI and waist circumference after these 8 years. As for sleep variables, we observed an increase in N1, reduction in N2, increase in the arousal index, the PLM, and the AHI, as depicted in Table 1.

**Table 1:** Descriptive analysis of the sample in baseline and follow-up.

	**Mean**		**Standard deviation**		**Variance**	
	**2007** ***N*** = **688**	**2015** ***N*** = **688**	**2007** ***N*** = **688**	**2015** ***N*** = **688**	**2007** ***N*** = **688**	**2015** ***N*** = **688**
Age (years)	42.20	50.10	13.38	13.36	179.24	178.64
BMI (kg/m^2^)	27.19	28.18	5.51	5.75	30.37	32.52
Neck circumference (cm)	36.27	37.50	4.77	4.18	22.84	17.54
Waist circumference (cm)	86.18	97.60	14.71	14.01	206.53	196.34
Hip circumference (cm)	101.40	104.20	11.98	11.08	143.45	124.80
Sleep latency (min)	15.15	15.40	20.62	22.10	425.41	464.63
REM sleep latency (min)	102.44	97.07	55.11	59.10	3038.07	3437.06
TST (min)	347.35	354.00	75.92	81.38	5763.91	6654.40
Sleep efficiency (%)	81.32	80.60	12.40	12.20	153.79	148.98
N1 (%)	4.96	14.00	3.48	10.50	12.17	12.09
N2 (%)	54.78	40.30	9.11	8.60	83.06	75.95
N3 (%)	21.50	24.90	7.61	9.10	58.01	83.14
REM sleep (%)	19.01	20.60	6.76	7.20	45.80	53.39
Arousal index (event/hour)	15.49	22.50	11.65	12.90	135.95	168.62
PLM index (event/hour)	3.56	4.94	9.99	11.90	99.73	146.97
AHI (event/hour)	8.50	17.00	13.62	18.30	185.70	339.88

BMI, body mass index; TST, total sleep time; PLM, periodic leg movement; AHI, apnea-hipopnea index.

We then carried the analysis stratifying the population into adult participants (2007: *n* = 612; 2015: *n* = 530) and older adults (2007: *n* = 76; 2015: *n* = 158). We created a category specifically to analyze the sleep behavior of those who reached the age of over 60 years at follow-up (new older adults), that is, a population aged 60 to 68 at follow-up (*n* = 82). After applying this classification, we allocated the participants in groups, according to sex, and compared their sleep parameters at baseline and follow-up. In 2007, the sample comprised 340 female adults vs. 272 male adults, and 49 female older adults vs. 27 male older adults. In 2015, the sample was composed by 289 female adults vs. 241 male adults, and 100 female older adults vs. 58 male older adults. Finally, we also included 51 new female older adults and 31 new male older adults at follow-up.

We noticed a higher BMI among male older adults (*p* = 0.043) at baseline, but at follow-up, new female older adults had higher BMI than male counterparts (*p* = 0.033). Men in every time period (2007–2015) and every age group showed higher neck circumference and waist circumference. For hip circumference, women stood out (Supplementary Table 1).

We found significant differences in sleep structure parameters between the sexes. REM latency was higher in female adults at baseline (*p* = 0.027) and remained higher at follow-up (*p* = 0.05). Sleep efficiency was higher in men at baseline but not at follow-up when women had higher values (*p* = 0.041). Men had a higher percentage of N1 sleep in both periods, while women had a higher percentage of N3 (slow-wave sleep). The arousal index was higher in men in both periods. A higher PLM index was found in female older adults and new male older adults at baseline and in those who became older at follow-up (*p* = 0.03; *p* = 0.011; *p* = 0.05; and *p* = 0.014). The apnea/hypopnea index was higher among men in all age groups. No significant changes were reported for sleep latency, TST, N2, and REM sleep. The results are displayed in Supplementary Table 2.

We analyzed the interaction factor Sex^*^Time considering the PSG variables using Generalized Estimating Equations (GEE) model. Age and BMI were included in the model as covariates. We found a significant effect of the main factors for several variables. REM sleep latency differed according to sex (*p* = 0.010) with no significant interaction factor. TST, however, differed in time (*p* = < 0.001) also with no significant interaction. We found significant effects of sex, time, and interaction for sleep efficiency, N1, N3, arousal index, and AHI, as depicted in Table 2. We then used the Bonferroni Multiple Comparison (*Post-Hoc*) to compare the subgroups in pairs for those variables that reached significance in the interaction factor (sleep efficiency, N1, N3, arousal index, and AHI). A significant interaction remained for N1 (*p* = 0.001), N3 (*p* = 0.001), arousal index (*p* = 0.001), and AHI (*p* = 0.001).

**Table 2:** Generalized regression model considering sex and time and Interaction on polysomnography parameters after controlling for age and body mass index.

	**Sex**					**Time**					**Interaction**			
**Variables**	**Beta**	**95% confidence interval limits**		**Effect size**	**P-value**	**Beta**	**95% confidence interval limits**		**Effect size**	**P-value**	**Beta**	**95% confidence interval limits**		**P-value**
Sleep latency (min)	0.901	−2.073	3.874	0.007	0.900	0.109	−2.970	3.188	0.043	0.394	−2.121	−6.092	1.851	0.295
REM sleep latency (min)	9.718	1.659	17.777	0.139	0.010^*^	−6.090	−14.392	2.213	0.126	0.151	−2.404	−13.154	8.347	0.661
TST (min)	−4.173	−14.994	6.648	0.019	0.726	14.986	4.325	25.648	0.267	< 0.001^*^	11.631	−1.792	25.053	0.089
Sleep efficiency (%)	−0.289	−2.017	1.438	0.082	0.131	−1.026	−2.452	0.400	0.031	0.486	2.794	0.840	4.748	0.005^*^
N1 (%)	−0.730	−1.314	−0.145	0.370	< 0.001^*^	10.630	9.274	11.987	1.112	< 0.001^*^	−4.344	−5.948	−2.739	< 0.001^*^
N2 (%)	−0.431	−1.809	0.948	0.042	0.439	−15.374	−16.804	−13.944	1.557	< 0.001^*^	1.678	−0.093	3.449	0.063
N3 (%)	1.463	0.336	2.590	0.258	< 0.001^*^	2.623	1.512	3.733	0.422	< 0.001^*^	1.989	0.542	3.436	0.007^*^
REM sleep (%)	−0.305	−1.319	0.709	0.005	0.934	2.117	1.192	3.042	0.350	< 0.001^*^	0.682	−0.543	1.906	0.275
Arousal index (%)	−4.996	−6.643	−3.348	0.459	< 0.001^*^	5.614	4.028	7.200	0.373	< 0.001^*^	−2.277	−4.088	−0.466	0.014^*^
PLM index (event/hour)	−0.172	−1.663	1.319	0.005	0.926	−0.315	−1.504	0.875	0.007	0.857	0.479	−1.090	2.047	0.550
AHI (event/hour)	−5.864	−7.627	−4.101	0.464	< 0.001^*^	6.361	4.302	8.420	0.308	< 0.001^*^	−3.467	−5.829	−1.104	0.004^*^

REM, rapid eye movement; TST, total sleep time; PLM, periodic leg movement; AHI, apnea-hipopnea index. ^*^P < 0.05 was considered significant.

**Figure 1:**
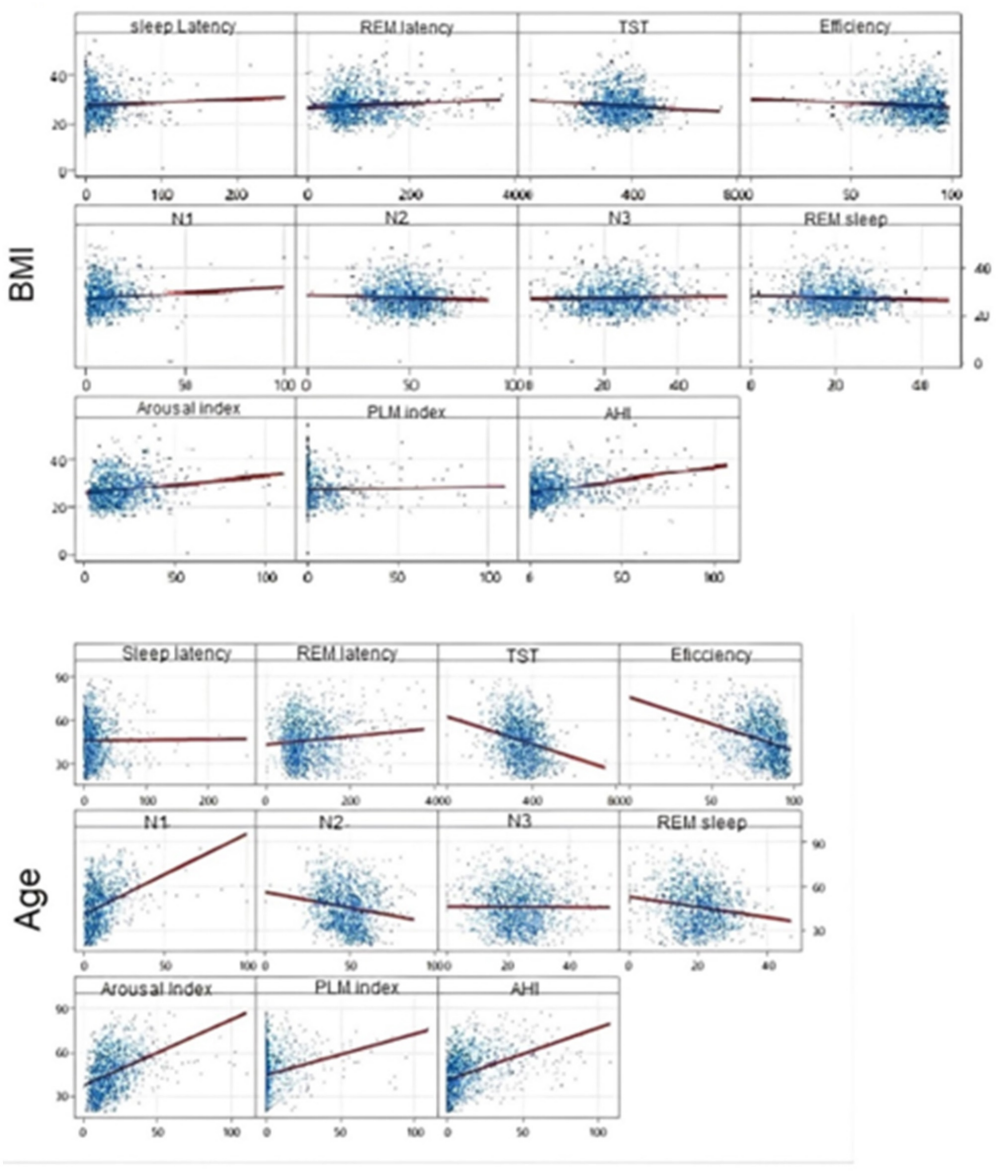
Scatter plot—Pearson's Correlation to assess the correlation between age and BMI with polysomnography parameters.

Finally, we used Pearson's Correlation to assess the correlation between age and BMI with polysomnography parameters. We found that age and BMI were significantly correlated with several sleep parameters, but the main values (with a correlation above 0.4) occurred for arousal index and AHI, but only with age, with positive correlations of *r* = 0.420 and *r* = 0.430, respectively (both with *p*-value < 0.001). The positive correlations suggest that the older the age, the greater the values of these two parameters and vice versa. The other correlations, although significant, were weak, as displayed in Figure 1.

## 4 Discussion

In this study, we assessed the influence of sex and age on sleep variables in a population-based sample of the city of São Paulo, Brazil. We evaluated the participants in 2007 and then again in 2015. We noticed increases in anthropometric parameters (BMI, neck and hip circumference), N1, awakenings, and the AHI, and a reduction in N2 and REM sleep. This may reflect the role of age in weight gain in the general population and is consistent with national and global data on obesity (National Heart Lung and Blood Institute, 2013). Research has shown that the proportion of obese people in the adult population has doubled in the last 15 years, and it is known that there is a difference in body fat distribution with aging and between sexes (De Pauw et al., 2022). Sleep disturbances, including short sleep, sleep debt, and circadian misalignment, may also disrupt glucose homeostasis and cause metabolic perturbations via other hormonal and cellular signaling cascades and this can be a 2-way street where obesity interferes with sleep and poor sleep may cause obesity (Ding et al., 2018).

Previous studies in the literature have reported worse subjective sleep quality in older women (Fukuda et al., 1999), although PSG data showed better sleep structure compared to men (Reyner et al., 1995). This is, to some extent, consistent with the results of this study, with PSG indicating that adult women had higher sleep efficiency and longer time in SWS compared to men, while men had more superficial sleep than women, as seen by the higher amount of stage N1 sleep with a higher arousal index for every age in both periods (2007 and 2015; Mourtazaev et al., 1995; Rediehs et al., 1990).

Although there are recognized sex differences concerning hormonal profiles, body fat composition, physical attributes, and even susceptibility to sleep disorders, the disparity in sleep perception between the sexes remains to be elucidated. We found a higher PLM index in older women and a predominance of OSA among men. It is known that age itself is an important factor in the prevalence of both of these conditions (Ohayon and Roth, 2002; Scofield et al., 2008), but our findings in respect to their association with sex are in line with the literature. Some studies suggest that PLM is more prevalent in older women; however, the sex-specific prevalence among older populations is not consistently reported. One study found that older age, women, shift work, stress, and caffeine intake were risk factors for PLM and restless legs syndrome (Allen et al., 2005). It has also been reported that insomnia was significantly higher in individuals with a PLM > 15 (Innes et al., 2011).

While insomnia and PLM seem to be more frequent in women, OSA tends to be more common in men. A recent cohort study of, 1,010 Greek patients diagnosed with OSA showed that this disorder was 5 times more common in men (Vagiakis et al., 2006). Several studies confirm that OSA affects men more, but they point out that women may be underdiagnosed and that interference of age must be considered since OSA in women tends to appear later in life (da Daltro et al., 2006).

Longitudinal studies showed that sleep changes were mainly noticeable before the age of 60 years old. Studies have correlated SWS with age, BMI, and AHI together (Li et al., 2020). The question arises as to whether these differences reflect neuroanatomical differences rather than gender differences in sleep regulatory mechanisms (Pfefferbaum and Rosenbloom, 1987; Dijk et al., 1989; Dijk, 2009). However, we also acknowledge that other potential factors can affect sleep homeostatic process. Disrupted breathing can fragment sleep, preventing deep and restful sleep, possibly resulting in a decrease in sleep efficiency, total sleep time, percentages of N3, and REM sleep (Kim et al., 2021). The sex-related differences in sleep architecture should not be driven only by sleep apnea but by a set of variables such as age, anthropometric indicators, emotional characteristics, lifestyle factors and comorbidities. A thorough investigation of sleep behavior evolution in middle-aged and older adult populations stratified by age group and sex is needed to provide a coherent interpretation of the consequences of inadequate sleep, considering the substantial inter-individual variability.

This study has some limitations that should be mentioned. Firstly, we did not properly screen for restless legs syndrome. Secondly, participants only underwent a single night of full in-lab PSG recording with a very low refusal rate. Menopause assessment has not been done, however as we included a population over 60 years old, it is probably unlikely women participants are still pre-menopause. However, we also acknowledge that hormonal treatment can affect sleep patterns, which has not been assessed in the present study.

In conclusion, this manuscript prospectively and longitudinally evaluated, with full in-lab PSG, the sleep of the general population and reported objective findings according to sex and age. Our results suggest that sleep changes differently with the years for males and females. Understanding the mechanisms behind these changes among men and women can help targeting prevention, individual treatment, and better sleep quality.

## Data Availability

The datasets presented in this article are not readily available due to concerns regarding participant anonymity. Requests to access these datasets should be directed to mayra.naloto@gmail.com.

## Author disclaimer

All authors confirm that this work contains no libelous or unlawful statements and does not infringe or violate the public or privacy rights of any third party. It does not libel or slander any third party, or contains scandalous, obscene, or negligibly prepared information, or infringe or violate any other personal or proprietary right of others. Each author also confirms that all statements contained in the work purporting to be facts are true, and any formula or instruction contained in the work will not, if followed accurately, cause any injury, illness, or damage to the user.
