# Supplementary figures and images for: The influence of sex on sleep characteristics in the adult and older adult population: findings from the EPISONO sleep study
Source: Front Sleep. 2025 Feb 10;4:1422169. doi: 10.3389/frsle.2025.1422169 (PMC12713917; doi:10.3389/frsle.2025.1422169)

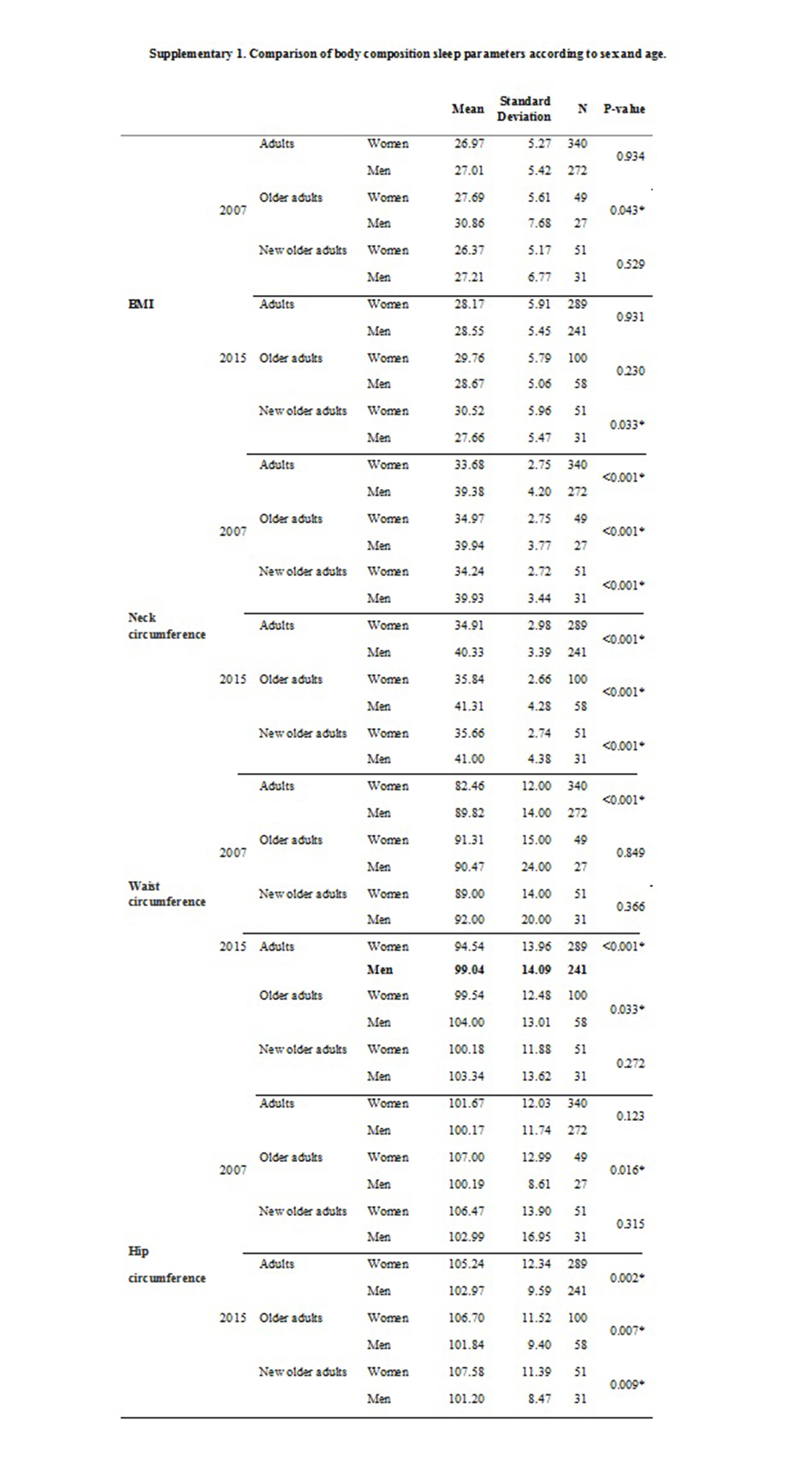

Supplement: Supplementary file 1 [file Image_1.jpeg]

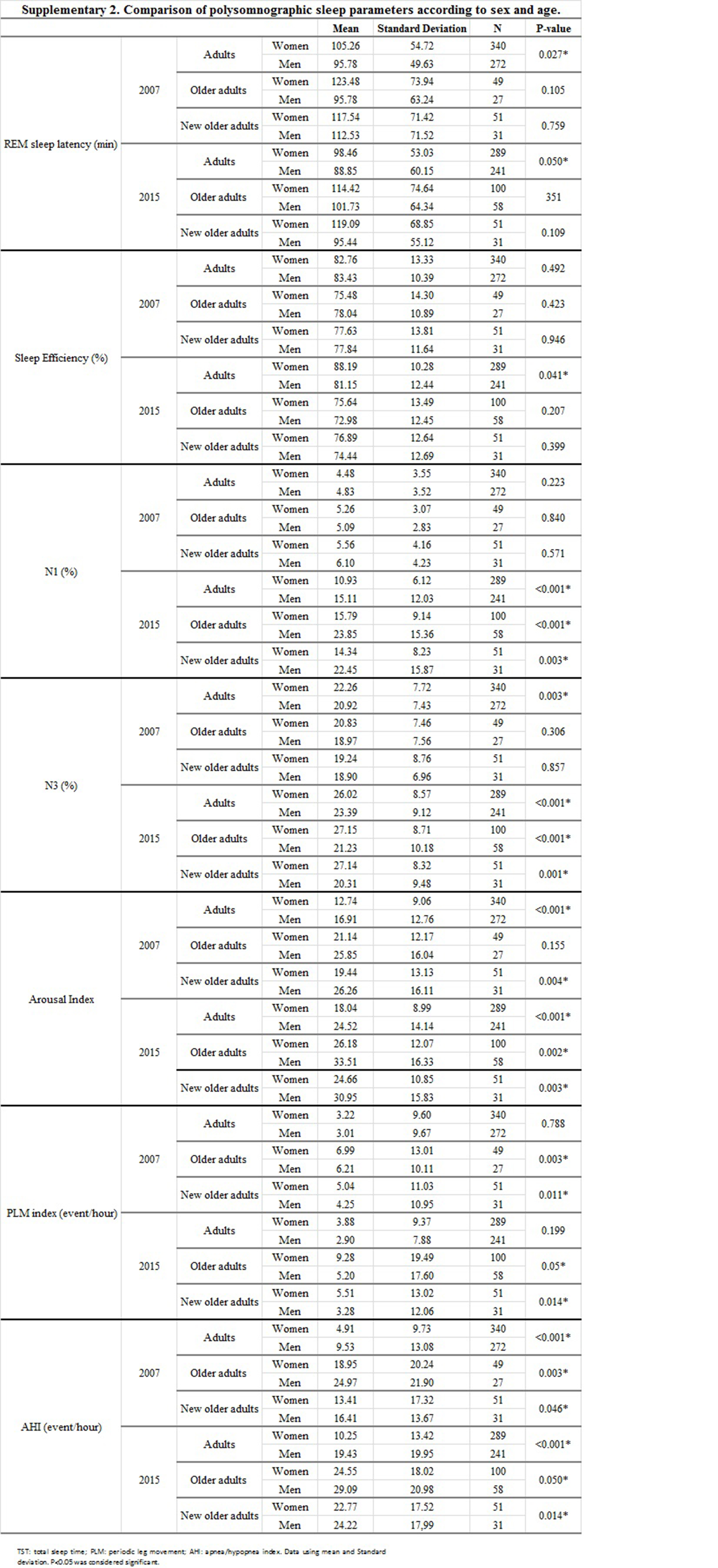

Supplement: Supplementary file 2 [file Image_2.jpeg]
